# Genetic polymorphisms and pancreatic cancer risk: A PRISMA-compliant systematic review and meta-analysis

**DOI:** 10.1097/MD.0000000000016541

**Published:** 2019-08-09

**Authors:** Peng Dai, Jing Li, Weibin Li, Xueliang Qin, Xiaoyong Wu, Weidong Di, Yanzhong Zhang

**Affiliations:** a Department of Hepato-Biliary-Pancreatic Surgery, Shanxi Provincial People's Hospital Affiliated to Shanxi Medical University; b Department of Microbiology and Immunology, School of Basic Medical Sciences, Shanxi Medical University; c Department of General Surgery, Shanxi Academy of Medical Sciences, Shanxi Dayi Hospital, Taiyuan, Shanxi, China.

**Keywords:** genetic polymorphisms, meta-analysis, pancreatic cancer

## Abstract

**Backgrounds:**

Previous investigations yielded inconsistent results for the associations between pancreatic cancer (PC) risk and genetic polymorphisms. The study aimed to perform a systematic review and meta-analysis of studies exploring association of some genetic polymorphisms and PC risk.

**Methods:**

We systematically searched on PubMed and Web of Science for association of genetic polymorphisms and PC risk published from 1969 to January 2019. We computed the multivariate odd ratio (OR) and 95% confidence intervals (CI), comparing different genetic types.

**Results:**

The present meta-analysis showed significant associations between deoxyribonucleic acid (DNA) repair gene (X-ray repair cross-complementing group 1 (XRCC1) *Arg399GIn* and *Arg194Trp*, excision repair cross complementation 1 (ERCC1) rs11615 and rs3212986, ERCC2 rs13181) polymorphisms and PC risk.

**Conclusions:**

Because of the limited sample size and ethnicity enrolled in the present meta-analysis, further larger scaled studies should be performed to demonstrate the association.

## Abstract 2

Supplemental Digital Content is available in the text

## 1 Introduction

Pancreatic cancer (PC) is one of the most fatal malignant tumors.^[1]^ The five-year survival rate remains as low as 6% even after the surgical and chemotherapy intervention.^[2,3]^ The late stage at which most patients are diagnosed might be one of the most important factors contributing to the low survival rate. But there is still no standard program for screening patients at high risk of PC. It is known that the development of PC is a complex and multifactorial process. Many factors, such as smoking, drinking, diabetes, obesity, body mass index, as well as environmental chemicals, are known to play a key role in PC development.^[4,5]^ Nevertheless, hereditary factors could not be ignored and might play an essential role in PC development. Emerging evidence suggests that the human genes for metabolism (cytochrome P-450 1A1 (CYP1A1), glutathione S-transferase M1 (GSTM1), glutathione S-transferase T1 (GSTT1),^[6]^ N-acetyltransferase 2 (NAT2),^[7]^ UDP glucuronosyltransferase (UGT1A7),^[8]^ methylation gene (Methylenetetrahydrofolate reductase (*MTHFR*)),^[9]^ inflammatory response gene (tumour necrosis factor (TNF)-α),^[10]^ deoxyribonucleic acid (DNA) repair gene (X-ray repair cross-complementing group 1 gene (*XRCC1*),^[11]^ 8-oxoguanineDNA glycosylase (OGG)1,^[12]^ excision repair cross complementation (ERCC) 1, ERCC2),^[13]^ alcohol-metabolizing enzyme gene (aldehyde dehydrogenase (ALDH) 2),^[14]^ and another gene (Kazal type 1 serine protease inhibitor (SPINK1))^[15]^ are most important candidate genes for influencing the risk of PC, and genetic polymorphisms in these gene might be associated with PC risk. However, previous investigations yielded inconsistent results for association of these genetic polymorphisms and PC risk. In the present investigation, we aim to perform a systematic review and meta-analysis to evaluate the association of these genetic polymorphisms and PC risk. In addition, we conducted subgroup studies according to different ethnicities.

## 2 Methods

This study was conducted on the basis of Preferred Reporting Items for Systematic Reviews and Meta-Analyses (PRISMA) statement ^[16]^. We supplied a PRISMA 2009 checklist. Ethical approval was not applicable in the study.

### 2.1 Search strategy

Articles on genetic polymorphism and PC risk were searched for in PubMed and Web of Science databases until January 2019. Search terms were the following: (“pancreatic cancer” OR “pancreatic carcinoma” OR “pancreatic neoplasm”) AND (“gene” OR “polymorphisms”). After that, duplicates were removed. A total of 71 articles were screened in our study.

### 2.2 Inclusion criteria and exclusion criteria

Our study included all articles exploring an association between gene polymorphisms and PC risk in adult humans. The study included in the study should be a case-control or cohort study. Additionally, studies should include both controls and PC patients as participants. All included studies reported odds ratio (OR) or data from which OR could be calculated. Moreover, there were no restrictions on language.

Articles were eliminated while they were associated endocrine neoplasms of the pancreas, familial PC, and hereditary PC syndrome. Secondary processing of literature such as reviews and meta-analysis articles were dropped. Case studies without group-level statistics were excluded.

### 2.3 Data collection

Titles and abstracts of articles were read by 2 different individuals. According to the inclusion and exclusion criteria, 71 articles were selected to read full-texts. We recorded following data from these full-texts: Author, publication years, country of origin, ethnicity, gene polymorphisms studied, mean age (standard deviation (SD)) of cases and controls, numbers of case and control populations, pathology of diseases, the exact genotyping techniques, genotyping quality control measures, evidence of the Hardy–Weinberg equilibrium (HWE), and variables for which statistical adjustments were made.

### 2.4 Meta-analysis for studies

We conducted meta-analysis to summarize results while at least 3 articles were presented for the strength of association between each polymorphism and PC risk. We computed the multivariate ORs and 95% CI. We assessed heterogeneity between studies with *Q* test, and evaluated the amount of variation derived from heterogeneity with computed *I*^2^. We conducted fixed effects models to generate summary effect size in absence of heterogeneity (*Q* test, *P* > .05) of included studies. Inversely, with invariably high heterogeneity between studies, we performed random effects models to summarize effect size. Subgroup analysis for different ethnicities was conducted to observe the effect of heterogeneities for ethnicities to the heterogeneity of meta-analysis. All statistical analysis was conducted with STATA 12.0 software.

## 3 Results

### 3.1 Search results

**Figure 1:**
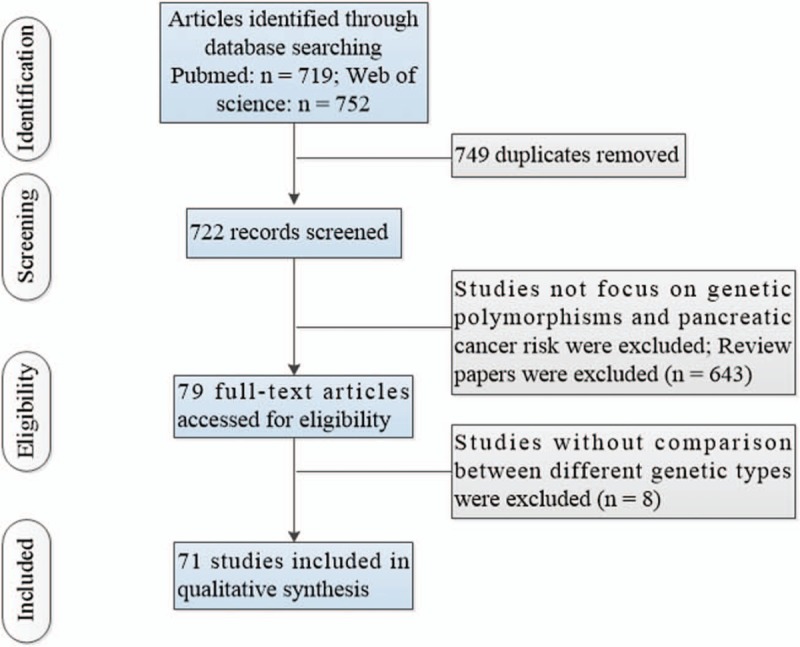
Flow of information through the different phases of a systematic review.

Figure 1 showed the flow chart of study exclusion and inclusion with specific reasons. Supplementary Table 1, http://links.lww.com/MD/D142 showed the study characteristics and results of the included 71 studies. We collected data from 6 XRCC1 (n = 1240 cases and 3918 controls),^[11,17–21]^ 5 OGG1 (n = 1714 cases and 3683 controls),^[12,17,22–24]^ 4 MTHFR (n = 957 cases and 1766 controls),^[9,25–27]^ 4 ERCC1 (n = 934 cases and 1039 controls)^[13,28–30]^ and 6 ERCC2 (n = 1759 cases and 2050 controls)^[13,23,28–31]^ studies.

### 3.2 Association between polymorphisms of DNA repair gene (*XRCC1*, *OGG1*, *ERCC1*, *ERCC2*) and PC risk

**Figure 2:**
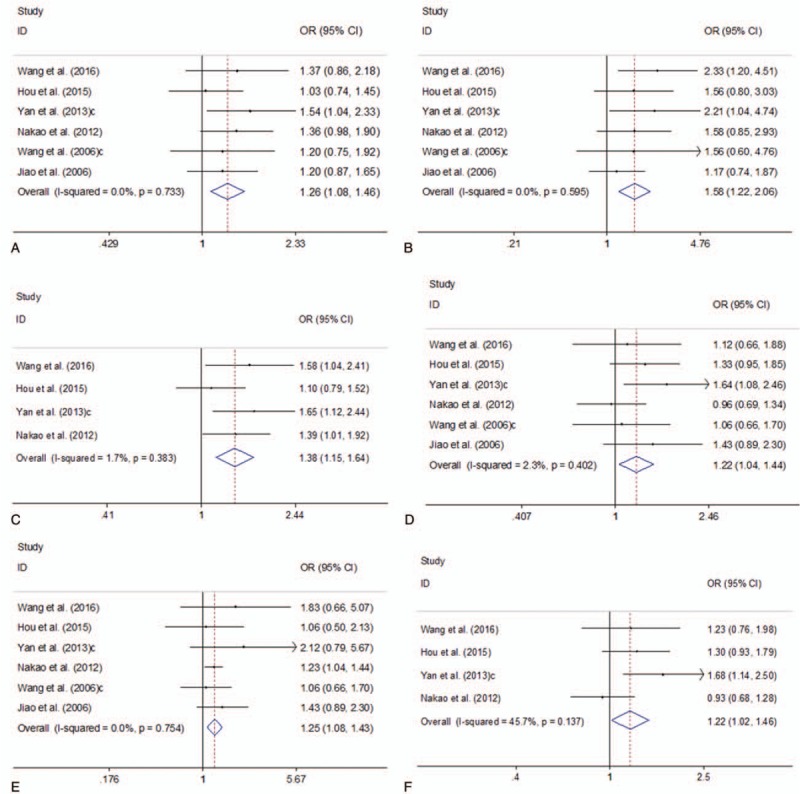
Forest plots of the association between XRCC1 Arg399GIn and XRCC1 Arg194Trp genetic polymorphisms and PC risk. A. XRCC1 Arg399GIn polymorphism (GA vs GG); B. XRCC1 Arg399GIn polymorphism (AA vs GG); C. XRCC1 Arg399GIn polymorphism (GA+AA vs GG); D. XRCC1 Arg194Trp polymorphism (CT vs CC); E. XRCC1 Arg194Trp polymorphism (TT vs CC); F. XRCC1 Arg194Trp polymorphism (CT+TT vs CC). CI = confidence interval, OR = odds ratio, PC = pancreatic cancer; XRCC1 = X-ray repair cross-complementing group 1 gene.

Six of the studies investigated XRCC1 Arg399GIn and XRCC1 Arg194Trp polymorphisms using 1240 cases and 3918 controls. Figure 2 showed the summary of this meta-analysis for the association strength between XRCC1 Arg399GIn and XRCC1 Arg194Trp genetic polymorphisms and PC risk. There were significant associations between XRCC1 Arg399Gin, Arg194Trp polymorphisms and PC risk in total population under all 3 genetic models (Arg399GIn: GA vs GG: OR = 1.26, 95%CI 1.08–1.46, *I*^2^ = 0.0%, *P* = .733; AA vs GG: OR = 1.58, 95%CI 1.22–2.06, *I*^2^ = 0.0%, *P* = .595; GA+AA vs GG: OR = 1.38, 95%CI 1.15–1.64, *I*^2^ = 1.7%, *P* = .383. Arg194Trp: CT vs CC: OR = 1.22, 95%CI 1.04–1.44, *I*^2^ = 2.3%, *P* = .402; TT vs CC: OR = 1.25, 95%CI 1.08–1.43, *I*^2^ = 0.0%, *P* = .754; CT+TT vs CC: OR = 1.22, 95%CI 1.02–1.46, *I*^2^ = 45.7%, *P* = .137) (Fig. 2). Further subgroup analysis by ethnicity indicated that there were statistically significant associations between XRCC1 Arg399GIn and Arg194Trp genetic polymorphisms and PC risk in Asians under all genetic models (all *P* values < .05, Supplementary Fig. 1, http://links.lww.com/MD/D142).

**Figure 3:**
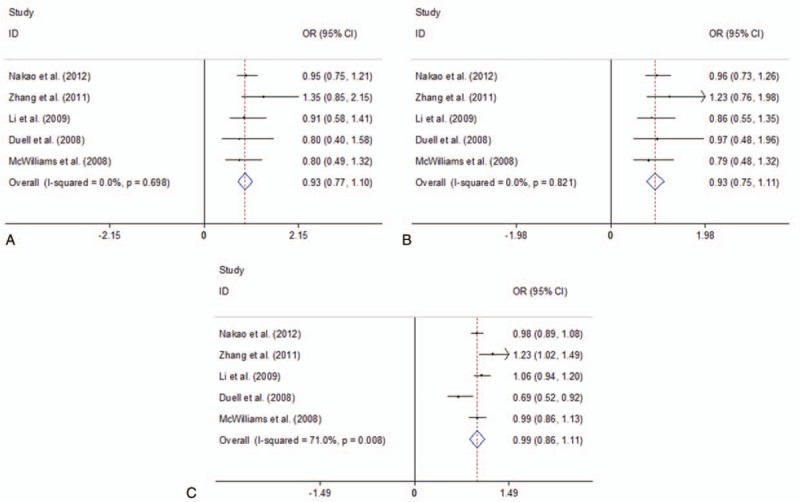
Forest plots of the association between OGG1 Ser326Cys polymorphism and PC risk. A. CC vs GG; B. CC vs GG+GC; C. CC+GC vs GG. CI = confidence interval, OR = odds ratio, PC = Pancreatic cancer; OGG1 = 8-oxoguanineDNA glycosylase 1.

Five of the studies explored OGG1 Ser326Cys polymorphism using 1714 cases and 3683 controls. Figure 3 presented the summary of this meta-analysis for the association strength between OGG1 Ser326Cys polymorphism and PC risk. There was no significant association between OGG1 Ser326Cys polymorphism and PC risk in total population under all 3 genetic models (CC vs GG: OR = 0.93, 95%CI 0.77–1.10, *I*^2^ = 0.0%, *P* = .698; CC vs GG+GC: OR = 0.93, 95%CI 0.75–1.11, *I*^2^ = 0.0%, *P* = .821; CC+GC vs GG: OR = 0.99, 95%CI 0.86–1.11, *I*^2^ = 71.0%, *P* = .008) (Figure 3). Further subgroup analysis by ethnicity indicated that there was no statistically significant association between OGG1 Ser326Cys polymorphism and PC risk in Caucasians under all genetic models (all *P* values > .05, Supplementary Fig. 2, http://links.lww.com/MD/D142).

**Figure 4:**
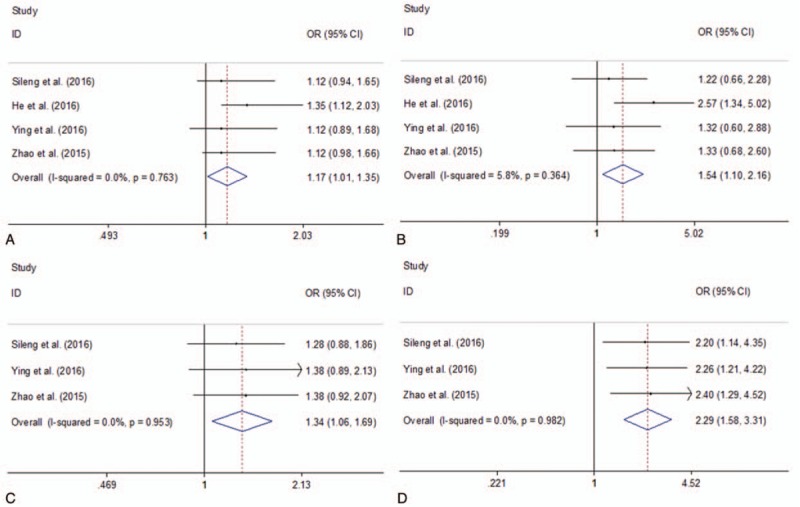
Forest plots of the association between ERCC1 rs11615 and rs3212986 polymorphisms and PC risk. A. ERCC1 rs11615 polymorphism (CT vs CC); B. ERCC1 rs11615 polymorphism (TT vs CC); C. ERCC1 rs3212986 polymorphism (GT vs GG); D. ERCC1 rs3212986 polymorphism (TT vs GG). CI = confidence interval, OR = odds ratio, PC = pancreatic cancer; ERCC1, excision repair cross complementation 1.

Four studies investigated ERCC1 rs11615 polymorphism using 934 cases and 1039 controls. Three studies investigated ERCC1 rs3212986 polymorphism using 717 cases and 795 controls. Figure 4 presented the summary of this meta-analysis for the association strength between ERCC1 rs11615 and rs3212986 polymorphisms and PC risk. There were significant associations between ERCC1 rs11615 and rs3212986 polymorphisms and PC risk in total population under all 2 genetic models (ERCC1 rs11615: CT vs CC: OR = 1.17, 95%CI 1.01–1.35, *I*^2^ = 0.0%, *P* = .763; TT vs CC: OR = 1.54, 95%CI 1.10–2.16, *I*^2^ = 5.8%,  = .364; ERCC1 rs3212986: GT vs GG: OR = 1.34, 95%CI 1.06–1.69, *I*^2^ = 0.0%,  = .953; TT vs GG: OR = 2.29, 95%CI 1.58–3.31, *I*^2^ = 0.0%,  = .982) (Figure 4).

**Figure 5:**
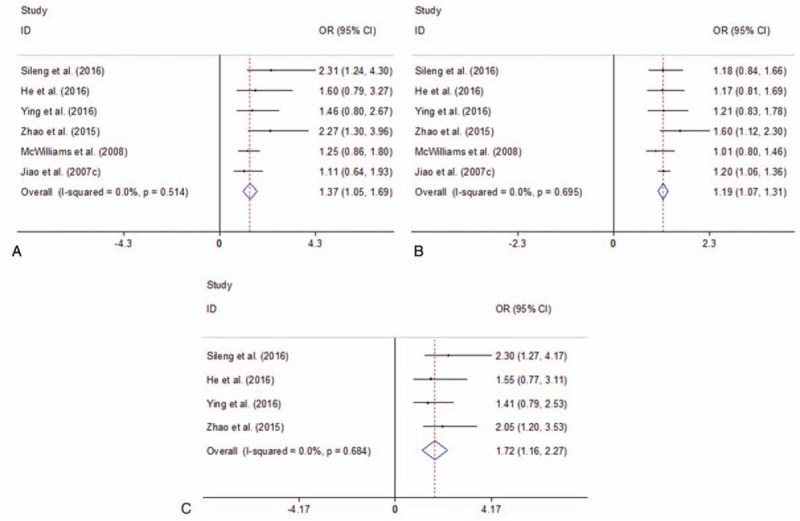
Forest plots of the association between ERCC2 rs13181 polymorphism and PC risk. A. CC vs AA; B. AC/CC vs AA; C. CC vs AC/CC. CI = confidence interval, OR = odds ratio, PC = pancreatic cancer, ERCC2, excision repair cross complementation 2.

Four studies investigated ERCC2 rs13181 polymorphism using 1759 cases and 2050 controls. Figure 5 presented the summary of this meta-analysis for the association strength between ERCC2 rs13181 polymorphism and PC risk. There was a significant association between ERCC2 rs13181 polymorphism and PC risk in total population under all 3 genetic models (CC vs AA: OR = 1.37, 95%CI 1.05–1.69, *I*^2^ = 0.0%,  = .514; AC/CC vs AA: OR = 1.19, 95%CI 1.07–1.31, *I*^2^ = 0.0%,  = .695; CC vs AC/CC: OR = 1.72, 95%CI 1.16–2.27, *I*^2^ = 0.0%, *P* = .684) (Fig. 5). Further subgroup analysis by ethnicity indicated that there was a statistically significant association between ERCC2 rs13181 polymorphism and PC risk in Asians under all genetic models (all *P* values < .05, Supplementary Fig. 3, http://links.lww.com/MD/D142).

### 3.3 Association between polymorphisms of Methylation gene (*MTHFR*) and PC risk

**Figure 6:**
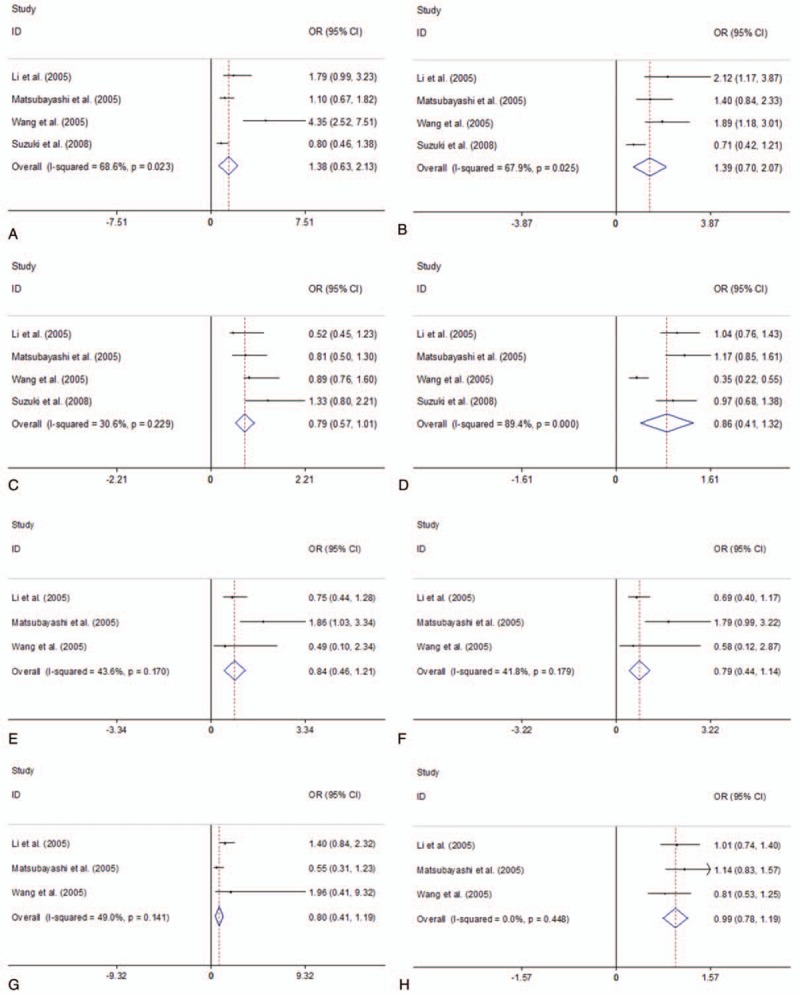
Forest plots of the association between MTHFR C677T and A1298C polymorphisms and PC risk. A. MTHFR C677T polymorphism (TT vs CC); B. MTHFR C677T polymorphism (TT vs CT); C. MTHFR C677T polymorphism (TT+ CT vs CC); D. MTHFR C677T polymorphism (TT vs CT+CC); E. MTHFR A1298C polymorphism (TT vs CC); F. MTHFR A1298C polymorphism (TT vs CT); G. MTHFR A1298C polymorphism (TT+ CT vs CC); H. MTHFR A1298C polymorphism (TT vs CT+CC). CI = confidence interval, OR = odds ratio; PC = pancreatic cancer; MTHFR = Methylenetetrahydrofolate reductase.

Four of the studies explored MTHFR C677T and A1298C polymorphisms using 957 cases and 1766 controls. Figure 6 presented the summary of this meta-analysis for the association strength between MTHFR C677T and A1298C polymorphisms and PC risk. There were no significant association between MTHFR C677T and A1298C polymorphisms and PC risk in total population under all 4 genetic models (MTHFR C677T: TT vs CC: OR = 1.38, 95%CI 0.63–2.13, *I*^2^ = 68.6%, *P* = .023; TT vs CT: OR = 1.39, 95%CI 0.70–2.07, *I*^2^ = 67.9%, *P* = .025; TT+ CT vs CC: OR = 0.79, 95%CI 0.57–1.01, *I*^2^ = 30.6%, *P* = .229; TT vs CT+CC: OR = 0.86, 95%CI 0.41–1.32, *I*^2^ = 89.4%, *P* < .001; MTHFR A1298C: TT vs CC: OR = 0.84, 95%CI 0.46–1.21, *I*^2^ = 43.6%, *P* = .170; TT vs CT: OR = 0.79, 95%CI 0.44–1.14, *I*^2^ = 41.8%, *P* = .179; TT+ CT vs CC: OR = 0.80, 95%CI 0.41–1.19, *I*^2^ = 49.0%, *P* = .141; TT vs CT+CC: OR = 0.99, 95%CI 0.78–1.19, *I*^2^ = 0.0%, *P* = .448) (Fig. 6).

## 4 Discussion

The present meta-analysis result showed significant associations between DNA repair gene (XRCC1 Arg399GIn and Arg194Trp, ERCC1 rs11615 and rs3212986, ERCC2 rs13181) polymorphisms and PC risk. In addition, further subgroup analysis by ethnicity indicated that there was a statistically significant association between XRCC1 Arg399GIn and Arg194Trp, ERCC2 rs13181 polymorphisms and PC risk in Asians under all genetic models.

The present study showed significant associations between DNA repair gene polymorphisms (XRCC1 Arg399GIn and Arg194Trp, ERCC1 rs11615 and rs3212986, ERCC2 rs13181) and PC risk. On the basis of genetic profiles of PC, genomic instability mediated by DNA repair deficiency is a crucial event in development of PC. DNA repair machinery plays an important role in defending cells against environmental hazards like ultraviolet (UV) rays, ionizing radiation, diet, and smoking. A key DNA repair mechanism, nucleotide excision repair (NER), can have an impact on gene–gene rearrangement, deletion, translocation, and amplification.^[32,33]^ NER pathway could identify the site of damage, unwind the DNA duplex around the site, cut the DNA upstream and downstream of the damaged area, and repair the gap.^[34,35]^ The influence of NER gene polymorphisms on PC risk is not quite well studied. In the present study, polymorphisms of 3 NER genes investigated (XRCC1, ERCC1 and ERCC2) were demonstrated to be associated with PC risk. The XRCC1 is located on chromosome 19q13.2-13.3.^[36]^ XRCC1 shows a variety of single nucleotide polymorphisms, of which those in the tenth and sixth exons are common and lead to Arg399GIn and Arg194Trp amino acid substitutions, respectively. The present study showed a result distinct from a recent meta-analysis, which suggests that the XRCC1 Arg194Trp genetic polymorphism is not significantly associated with PC risk.^[37]^ In these included studies, Jiao et al indicated no significant associations between XRCC1 Arg194Trp polymorphism and PC risk.^[31]^ Wang et al reported no significant differences in PC risk in participants with different XRCC1 Arg194Trp genetic polymorphism.^[20]^ Nakao et al observed no significant associations between PC risk and XRCC1 Arg194Trp polymorphism.^[17]^ In addition, Hou et al showed no significant relation between XRCC1 Arg194Trp polymorphism and PC risk.^[19]^ Wang et al reported that Arg194Trp polymorphisms did not affect PC risk.^[18]^ However, Yan et al indicated that the XRCC1 Arg194Trp genetic polymorphism might be associated with the risk of PC. ^[11]^ The result of the present meta-analysis might be influenced by the article published by Yan et al. The present study included further larger scaled studies, which caused the inconsistent result. *ERCC1* and *ERCC2* are DNA repair genes with the chromosomal locus 19q13.3. They could reverse ionizing radiation-induced damage and DNA damage by chemotherapy.^[38,39]^ A recent study indicated that ERCC2 rs13181 polymorphism might be important in stimulating the development of PC, especially for Asians.^[40]^ Our result was consistent with the study. He et al indicated no significant associations between ERCC2 rs13181 polymorphism and PC risk.^[13]^ Ying et al reported no significant associations between ERCC2 rs13181 polymorphism and PC risk. ^[29]^ However, Sileng et al indicated that ERCC2 rs13181 polymorphism exposed higher risk to PC.^[30]^ In addition, McWilliams et al indicated that ERCC2 rs13181 polymorphism was associated with PC risk. ^[23]^ Zhao et al showed that *ERCC2 rs13181* gene polymorphisms contribute to the development of PC. ^[28]^ The inconsistent results from different articles could not be explained by the ethnicity, research type or numbers of participants. But the present study provided a chance to summary the results in these articles. In addition, the summarized result is consistent with other meta-analysis. ^[40]^

The present study showed no significant association between MTHFR polymorphism and PC risk. MTHFR is a crucial enzyme within the folate methionine pathway. Folate intake increases plasma folate and reduces total homocysteine concentration, which may reduce and risk of cancer. In addition, the possible mechanism for the effect of MTHFR on PC is that DNA methylation, which might be associated with PC risk.^[41,42]^ These included studies showed inconsistent results, which might be caused by different folate status of ethnic differences and the environment in which they lived in. Result of the present study was corresponding to a recent study, which indicated that MTHFR polymorphisms (C667T and A1298C) are not associated with PC risk.^[43]^

This study showed significant associations between XRCC1 Arg399GIn and Arg194Trp genetic polymorphisms and PC risk in Asians, whereas Jiao et al indicated that no significant associations between XRCC1 Arg399GIn and Arg194Trp genetic polymorphisms and PC risks in Caucasians.^[21]^ Considering the limited sample size enrolled in this meta-analysis, further larger scaled studies are essential to provide a more precise estimation on the association in Caucasians. Subgroup study indicated no significant associations between OGG1 Ser326Cys polymorphism and PC risk in Caucasians. In addition, Nakao et al indicated no significant associations between OGG1 Ser326Cys polymorphism and PC risk in Asians.^[17]^ Subgroup study indicated significant associations between ERCC2 rs13181 polymorphism and PC risk in Asians, whereas the associations were not obvious between ERCC2 rs13181 polymorphism and PC risk in Caucasians. The differences between Asians and Caucasians may be partly result from the different genetic backgrounds and environments or lifestyles. The result is corresponding to a recent meta-analysis. ^[40]^

There were some limitations in the study. Firstly, the present study only included case-control studies. No prospective cohort studies were included in the study. Second, only 7 studies had a population-based design in the selection of cases. Most of the studies were from hospitals.

## 5 Conclusions

In conclusion, the present meta-analysis suggested significant associations between DNA repair gene (XRCC1 Arg399GIn and Arg194Trp, ERCC1 rs11615 and rs3212986, ERCC2 rs13181) polymorphisms and PC risk. Because of the limited sample size and ethnicity enrolled in the present meta-analysis, further larger scaled studies should be performed to demonstrate the association.

## Author contributions

**Data curation:** Peng Dai, Jing Li, Weibin Li, Xueliang Qin, Xiaoyong Wu, Weidong Di, Yanzhong Zhang.

**Methodology:** Peng Dai, Xueliang Qin, Xiaoyong Wu, Weidong Di.

**Writing** – **original draft:** Peng Dai.

**Writing** – **review & editing:** Peng Dai.

**Investigation:** Jing Li, Weibin Li.

**Software:** Yanzhong Zhang.

## Supplementary Material

Supplemental Digital Content
