# Supplementary material for: Genetic polymorphisms and pancreatic cancer risk: A PRISMA-compliant systematic review and meta-analysis
Source: Medicine (Baltimore). 2019 Aug 9;98(32):e16541. doi: 10.1097/MD.0000000000016541 (PMC6708677; doi:10.1097/MD.0000000000016541)
Supplement: Supplemental Digital Content [file medi-98-e16541-s001.docx]

Supplementary table 1 Characteristics of studies that evaluated the association of pancreatic cancer with gene polymorphisms

| Study (years) | Place of study | Genes  studied | Ethnic  background | Mean age  (SD) of  cases/  controls | No. of  cases/  controls | Diagnosis of cases  (no. of patients)^a^ | Representativeness  of  cases | Type of controls | Source of  control  selection | Genotyping  technique | HWE P valueb | Covariates |
| --- | --- | --- | --- | --- | --- | --- | --- | --- | --- | --- | --- | --- |
| Wu et al. (2017)[^1^](#_ENREF_1) | China | STAT3 | Asian | 61/60 | 940/1398 | Pathologically | University  hospital | Healthy individuals | Hospital-based | NR | 0.05 | sex and  age |
| Wang et al. (2017)[^2^](#_ENREF_2) | China | CTNND1 755 | Asian | 69.6(7.3)/ 63.2(8.1) | 122/180 | Pathologically | University  hospital | Healthy individuals | Hospital-based | PCR | 0.05 | age and gender |
| Hu et al. (2017)[^3^](#_ENREF_3) | China | HOTTIP, HOTAIR, H19 | Asian | NR | 921/921 | Pathologically | University  hospital | Healthy individuals | Hospital-based | PCR | 0.099HOTTIP,  0.166HOTAIR,  0.302  H19 | age, gender, smoking status, alcohol intake, body  mass index, hypertension, and history of pancreatitis,  diabetes mellitus, and cancer |
| Wang et al. (2016)[^4^](#_ENREF_4) | China | XRCC1 Arg399Gln,Arg280His, Arg194Trp | Asian | NR | 152/264 | Pathologically | University  hospital | Healthy individuals | Hospital-based | PCR-RFLP | NR | gender and age |
| Hussain et al. (2016)[^5^](#_ENREF_5) | Pakistan | CYP1A1 | Mixed | 66.8(11.1)/ 61.8(9.8) | 255/320 | Pathologically | University  hospital | Healthy individuals | Hospital-based | PCR-RFLP | NR | age, family history, smoking and sex |
| Sileng et al. (2016)[^6^](#_ENREF_6) | China | ERCC1  ERCC2 | Asian | 60.50(8.56)/59.65(9.47) | 254/277 | Pathologically | University  hospital | Healthy individuals | Hospital-based | PCR-  RFLP | NR | Age, gender |
| He et al. (2016)[^7^](#_ENREF_7) | China | ERCC1  ERCC2 | Asian | 64.64(11.53)/65.65(10.74) | 217/244 | Pathologically | the Nuclear Industry 215  Hospital | Healthy individuals | Hospital-based | PCR-RFLP | 0.38  ERCC1  0.17  ERCC2 | gender, age, smoking tobacco, alcohol consumption, and BMI |
| Ying et al. (2016)[^8^](#_ENREF_8) | China | ERCC1, ERCC2, ERCC3, ERCC4, ERCC5 | Asian | NR | 217/272 | Pathologically | the Nuclear Industry 215  Hospital | Healthy individuals | Hospital-based | PCR | NR | gender, age, and tobacco smoking and alcohol drinking habits |
| Zhao et al. (2015)[^9^](#_ENREF_9) | China | ERCC1, ERCC2, ERCC3, ERCC4, ERCC5, XPA, XPC, DDB2 | Asian | NR | 246/246 | Pathologically | the Nuclear Industry 215  Hospital | Healthy individuals | Hospital-based | PCR-RFLP | NR | sex, age, tobacco smoking, alcohol drinking, body mass index and family history of pancreatic cancer in the first  relatives |
| Ueno et al. (2015)[^10^](#_ENREF_10) | Japan | NR5A2 | Asian | 67.8(8.8)/ 64.8 (9.5) | 360/400 | Pathologically | University  hospital | Healthy individuals | Hospital-based | NR | 0.64  rs3790843 0.54 rs3790844 | age, sex, body  mass index, diabetes, and cigarette smoking |
| Hou et al. (2015)[^11^](#_ENREF_11) | China | XRCC1 | Asian | 54.73(7.98) /54.24 (7.81) | 298/298 | Pathologically | University  hospital | Healthy individuals | Hospital-based | PCR-RFLP | NR | NR |
| Qin et al. (2015)[^12^](#_ENREF_12) | China | rs9904341 | Asian | 63.4(11.5)/ 62.6(10.7) | 261/224 | Pathologically | University  hospital | Healthy individuals | Hospital-based | PCR-RFLP | NR | gender, age, smoking status, diabetes status, and family history of cancer |
| Li et al. (2014)[^13^](#_ENREF_13) | China | VDR | Asian | 56.19(11.90)/ 57.47(11.73) | 633/385 | Pathologically | University  hospital | Healthy individuals | Hospital-based | PCR-RFLP | NR | age, gender, history of tobacco, history of alcohol, and diabetes mellitus |
| Zhao et al. (2014)[^14^](#_ENREF_14) | China | OGG1 | Asian | 56.76(15.43)/ 57.22(16.45) | 382/382 | Pathologically | University  hospital | Healthy individuals | Hospital-based | PCR-RFLP | NR | NR |
| Tian et al. (2014)^c^ [^15^](#_ENREF_15) | China | U2AF65 | Asian | 58.9(12.9)/60.3(12.8) | 711/1082 | Pathologically | University  hospital | Healthy individuals | Hospital-based | TaqMan | 0.185 | age, gender, history of tobacco, history of alcohol |
| Lu et al. (2014)[^16^](#_ENREF_16) | China | TRIB1 | Asian | 60.5(13.1)/ 52.9(14.6) | 182/359 | Pathologically | University  hospital | Healthy individuals | Hospital-based | NR | NR | NR |
| Liu et al. (2014)[^17^](#_ENREF_17) | China | CLPTM1L-TERT | Asian | NR | 766/821 | Pathologically | NR | Healthy individuals | NR | TaqMan | 0.263 | Age and gender |
| Chen et al. (2014)[^18^](#_ENREF_18) | China | OGG1 | Asian | 56.35(15.48) 58.36(16.32) | 347/364 | Pathologically | University  hospital | Healthy individuals | Hospital-based | PCR | NR | NR |
| Chen et al. (2013)[^19^](#_ENREF_19) | China | XRCC1 | Asian | 58.78(16.23)/59.78(17.18) | 328/350 | Pathologically | University  hospital | Healthy individuals | Hospital-based | PCR | NR | NR |
| Yan et al. (2013)^c^[^20^](#_ENREF_20) | China | XRCC1 | Asian | NR | 210/213 | Pathologically | University  hospital | Healthy individuals | Hospital-based | PCR | NR | NR |
| Xiang et al. (2012)[^21^](#_ENREF_21) | China | CD86 | Asian | 59.8(10.7)/61.3 (11.6) | 369/412 | Pathologically | University  hospital | Healthy individuals | Hospital-based | PCR–RFLP | NR | body mass index, smoking,  drinking, and diabetes status |
| Lang et al. (2012)[^22^](#_ENREF_22) | China | CTLA-4 | Asian | 54.1(16.7)/ 50.5(17.2) | 602/651 | Pathologically | University  hospital | Healthy individuals | Hospital-based | PCR–RFLP | >0.05 | NR |
| Nakao et al. (2012)[^23^](#_ENREF_23) | Japan | OGG1, XRCC1, APE1, PARP1 | Asian | NR | 185/1465 | Pathologically | University  hospital | Healthy individuals | Hospital-based | TaqMan | 0.321 | age, sex, current BMI, BMI at age 20, smoking  status, drinking habit, diabetes mellitus, and family history of PC |
| Willis et al. (2012)[^24^](#_ENREF_24) | USA | ABO, NR5A2, CLPTM1L-TERT | Caucasian | NR | 531/305 | Pathologically | University  hospital | Healthy individuals | Hospital-based | PCR | NR | age at diagnosis, sex, Karnofsky Performance Score, BMI and pancreatic cancer stage |
| Dong et al. (2012)[^25^](#_ENREF_25) | USA | IGF axis | All ethnicities | 62.6(10.0)/61.1(10.0) | 706/706 | Pathologically | University  hospital | Healthy individuals | Hospital-based | TaqMan | NR | sex, race, age, diabetes, smoking, alcohol consumption, and family history of cancer |
| Wang et al. (2011)[^26^](#_ENREF_26) | China | HIF-1 | Asian | 59(12)/59(11) | 263/271 | Pathologically | University  hospital | Healthy individuals | Hospital-based | PCR | NR | age, gender and smoking history |
| Rizzato et al. (2011)[^27^](#_ENREF_27) | Germany | ABO, SHH,  CLPTM1L-TERT, NR5A2 | Caucasian | NR | 690/1277 | Pathologically | University  hospital | Healthy individuals | Hospital-based | PCR | NR | age and gender |
| Li et al. (2011)[^28^](#_ENREF_28) | USA | SSTR5 | All ethnicities | 62.0(9.9)/ 61.5(9.7) | 863/876 | Pathologically | University  hospital | Healthy individuals | Hospital-based | PCR | NR | age, sex, and known risk factors such as family history of cancer, history of  diabetes, smoking status, and BMI |
| Sonoyama et al. (2011)[^29^](#_ENREF_29) | Japan | TP53 | Asian | NR | 226/448 | Pathologically | University  hospital | Healthy individuals | Hospital-based | PCR | NR | age,  gender, and history of smoking and drinking |
| Zhang et al. (2011)[^30^](#_ENREF_30) | USA | OGG1 | Caucasian | 65.9(11.2)/65.6 (12.4) | 189/486 | Pathologically | University  hospital | Healthy individuals | Hospital-based | PCR | NR | age, sex, race, education, cigarette  smoking, alcohol intake, physical activity, and energy intake. |
| Fong et al. (2010)[^31^](#_ENREF_31) | USA | CAPN10 | All ethnicities | NR | 83/166 | Pathologically | University  hospital | Healthy individuals | Hospital-based | PCR | NR | NR |
| Fei et al. (2010)[^32^](#_ENREF_32) | China | E-cadherine16 | Asian | NR | 254/101 | Pathologically | University  hospital | Healthy individuals | Hospital-based | PCR–RFLP | NR | sex and age |
| Naccarati et al. (2010)[^33^](#_ENREF_33) | Czech Republic | TP53 | NR | 62.2(10.4) /60.5(10.7) | 240/743 | Pathologically | University  hospital | Healthy individuals | Hospital-based | NR | NR | age and gender, smoking habit and body mass index |
| Petersen et al. (2010)[^34^](#_ENREF_34) | USA | CLPTM1L-TERT | All ethnicities | NR | 3851/3934 | Pathologically | University  hospital | Healthy individuals | Hospital-based | NR | NR | study, age, sex, self-described ancestry and five principal components of  population stratification |
| Duchonova et al. (2010)[^35^](#_ENREF_35) | Czech Republic | CYP2A13, ADH1B, ADH1C | Caucasian | 61.9(10.5) /57.9(10.6) | 235/265 | Pathologically | University  hospital | Healthy individuals | Hospital-based | PCR | NR | age at recruitment, sex,  weight, diabetes, pancreatitis, smoking, alcohol drinking, and  coffee and tea consumption |
| Vrana et al. (2009)[^36^](#_ENREF_36) | Czech Republic | GSTT1 | Caucasian | NR | 253/403 | Pathologically | University  hospital | Healthy individuals | Hospital-based | PCR | NR | age, sex, smoking status and education |
| Hamacher et al. (2009)[^37^](#_ENREF_37) | Germany | IL-1b | Caucasian | NR | 73/235 | Pathologically | University  hospital | Healthy individuals | Hospital-based | PCR-RFLP | NR | NR |
| Rafnar et al. (2009)[^38^](#_ENREF_38) | Spain | TERT- CLPTM1L | NR | NR | 525/515 | Pathologically | University  hospital | Healthy individuals | Hospital-based | PCR | NR | NR |
| Li et al. (2009)[^39^](#_ENREF_39) | USA | LIG3, LIG4, OGG1, ATM, POLB, RAD54L, RECQL | Caucasian | 62.2(9.6)/ 62.0(9.6) | 734/780 | Pathologically | University  hospital | Healthy individuals | Hospital-based | PCR | NR | smoking, alcohol, diabetes, family history of cancer among first-degree relatives |
| Suzuki et al. (2009)[^40^](#_ENREF_40) | USA | IGF | All ethnicities | NR | 892/783 | Pathologically | University  hospital | Healthy individuals | Hospital-based | PCR | NR | gender, race |
| Xu et al. (2008)^c^[^41^](#_ENREF_41) | China | COX-2 | Asian | 60(10)/60(11) | 283/566 | Pathologically | University  hospital | Healthy individuals | Hospital-based | PCR–RFLP | NR | Age and sex |
| Suzuki et al. (2008)[^42^](#_ENREF_42) | Japan | MTHFR, MTR, MTRR, TS | Asian | NR | 157/785 | Pathologically | University  hospital | Healthy individuals | Hospital-based | PCR | NR | age, sex, drinking habit, smoking habit, body mass index, total nonalcohol energy intake, dietary folate intake, history of diabetes mellitus,  and referral pattern to our hospital |
| Asomaning et al. (2008)[^43^](#_ENREF_43) | USA | MDM2 | All ethnicities | 63.7/64.5 | 123/372 | Pathologically | University  hospital | Healthy individuals | Hospital-based | TaqMan | NR | age, gender, smoking status, and pack-years of smoking |
| Duell et al. (2008)[^44^](#_ENREF_44) | France | OGG1,  XPD, XPA, XPC, XRCC3, XPD.751, XPD.312 | Caucasian | NR | 308/964 | Pathologically | University  hospital | Healthy individuals | Hospital-based | PCR-RFLP | NR | age, sex, and race or ethnicity |
| Ohnami et al. (2008)[^45^](#_ENREF_45) | Japan | MTRR | Asian | NR | 532/623 | Pathologically | University  hospital | Healthy individuals | Hospital-based | PCR | NR | age and smoking status |
| Zhang et al. (2008)[^46^](#_ENREF_46) | China | IL-6 | Asian | 63.6(12.2)/59.6(10.4) | 85/41 | Pathologically | University  hospital | Healthy individuals | Hospital-based | PCR | NR | sex, age,  duodenal obstruction, and CBD obstruction |
| Jiao et al. (2008)[^47^](#_ENREF_47) | USA | XRCC2, XRCC3 | Caucasian | NR | 498/468 | Pathologically | University  hospital | Healthy individuals | Hospital-based | PCR | NR | NR |
| Kanda et al.  (2008)[^48^](#_ENREF_48) | Japan | ALDH2 | Asian | NR | 160/1600 | NR | A district  hospital | Healthy outpatients  without cancer | Hospital-based | Taqman | 0.20 | Age, sex,  smoking,  diabetes,  BMI, and  family history  of pancreatic  cancer |
| McWilliams  et al. (2008)[^49^](#_ENREF_49) | USA | ERCC1, XPD/ERCC2, XPC, XPF/ERCC4, OGG1, XRCC1 | Caucasian | 65.7(10.5)/  59.7(12.1) | 481/625 | Pathologically | University  hospital | Healthy individuaals  with negative  results from  screening  colonoscopy for  colon cancer | Hospital-based | PCR | 0.53 (C/T) 0.52  (G/A) | NR |
| Jiao et al.  (2007a)[^50^](#_ENREF_50) | USA | GSTM1  GSTT1  GSTP1 | Caucasian | 61.7/61.4c | 352/315 | Pathologically | University  hospital | Healthy individuals  from friends and  spouses or in-laws  matched by age,  sex, and ethnicity | Hospital-based | PCR-RFLP  Masscode  system | 0.38  (GSTT1) | NR |
| Jiao et al.  (2007b)[^51^](#_ENREF_51) | USA | NAT1 NAT2 | Caucasian | NR | 532/581 | Pathologically | University  hospital | Healthy individuals  from friends and  spouses or in-laws  matched by age,  sex, and ethnicity | Hospital-based | Taqman | >0.15 | Age, sex,  smoking, and  diabetes |
| Jiao et al. (2007c)[^52^](#_ENREF_52) | USA | ERCC2 | Caucasian | NR | 344/386 | Pathologically | University  hospital | Healthy individuals  from friends and  spouses or in-laws  matched by age,  sex, and ethnicity | Hospital-based | PCR-RFLP | 0.001 | age and gender |
| Duell et al.  (2006)[^53^](#_ENREF_53) | USA | TNFa | Caucasian | NR | 260/860 | Pathologically | Populationbased  cancer  registry | Healthy individuals  matched in an  approximate 3 : 1  ratio by age and  gender | Population-based | PCR-RFLP  Masscode  system | 0.16 | Age and sex |
| Jiao et al.  (2006)[^54^](#_ENREF_54) | USA | XRCC1 | Caucasian | NR | 384/357 | Pathologically | University  hospital | Healthy individuals  from friends and  spouses or in-laws  matched by age,  sex, and ethnicity | Hospital-based | PCR-RFLP  Masscode  system | 0.57 | Age, sex, race,  and smoking |
| Piepoli et al.  (2006)[^55^](#_ENREF_55) | Italy | CFTR  SPINK1  UGT1A7 | Caucasian | 63(10)/  39(9) | 63(10)/  39(9) | Clinically (5)  Pathologically (59) | University  hospital | Healthy blood donors | Hospital-based | PCR-RFLP | >0.05 | NR |
| Wang et al.  (2006)c[^56^](#_ENREF_56) | China | XRCC1 | Asian | NR | 101/337 | Pathologically | University  hospital | Healthy individuals  matched by age  and sex | Population-based | PCR-RFLP | 0.79 | NR |
| Lempinen et al.  (2005)[^57^](#_ENREF_57) | Finland | SPINK1 | Caucasian | NR | 188/459 | Radiologically (82)  Pathologically (57) | University  hospital | Healthy blood donors | Hospital-based | PCR | NR | NR |
| Li et al. (2005)[^58^](#_ENREF_58) | USA | MTHFR | Caucasian | 62.1(10.3)/  59.9(11.5) | 304/311 | Pathologically | University  hospital | Healthy individuals  from family  members, spouses,  and friends  matched by age,  sex, and ethnicity | Hospital-based | PCR-RFLP | 0.13 | Age, smoking,  pancreatitis,  and diabetes |
| Matsubayashi  et al. (2005)[^59^](#_ENREF_59) | USA | MTHFR | All ethnicities | NR | 333/333 | Pathologically | University  hospital | Patients with benign  gallbladder  diseases matched  by age, sex, and  ethnicity | Hospital-based | PCR-RFLP | 0.80  (MTHFR677)  0.10  (MTHFR1298) | NR |
| Miyasaka et al.  (2005)[^60^](#_ENREF_60) | Japan | ALDH2 | Asian | NR | 114/2070 | Pathologically | A district  hospital | NR | Hospital-based | PCR-RFLP | 0.19 | NR |
| Verlaan et al.  (2005)[^61^](#_ENREF_61) | The  Netherlands | UGT1A7 | All ethnicities | 62(10)/  50(22) | 236/1409 | Pathologically | Hospitals from  Germany and  Switzerland | Healthy blood donors,  medical students  and staff and newborn  infants | Population-based | PCR-RFLP | >0.05 | NR |
| Wang et al.  (2005)[^62^](#_ENREF_62) | China | MTHFR | Asian | 61.3/59.5c | 163/337 | Radiologically (67)  Pathologically (96) | Two district  hospitals | Healthy individuals  matched by age  and sex | Population-based | PCR-RFLP | 0.27 | Age, sex,  smoking, and  drinking |
| Beranek et al.  (2003)[^63^](#_ENREF_63) | Germany | TNFa | Caucasian | NR | 208/116 | Pathologically | University  hospital | Healthy blood donors | Hospital-based | PCR | 0.30 | NR |
| Matsubayashi  et al. (2003)[^64^](#_ENREF_64) | USA | SPINK1 | 90%  Caucasian | NR | 200/117 | Pathologically | University  hospital | Patients with chronic  cholecystitis | Hospital-based | PCR-RFLP | NR | NR |
| Teich et al.  (2003)[^65^](#_ENREF_65) | Germany | SPINK1 | Caucasian | NR | 159/492 | Pathologically | Three district  hospitals | Healthy blood donors,  students, and staff | NR | NR | NR | NR |
| Duell et al.  (2002a)[^66^](#_ENREF_66) | USA | CYP1A1  GSTM1  GSTT1 | Caucasian | 21–85e | 309/964 | Pathologically | Population-based  cancer  registry | Healthy individuals  identified by  random digit dialing  and administration  lists matched by  age and sex | Population-based | PCR | (CYP1A1*2A)  0.003  (CYP1A1*2C)  0.72  (CYP1A1*4) | Age and sex |
| Duell et al.  (2002b)[^67^](#_ENREF_67) | USA | XRCC1 | Caucasian | 21–85e | 309/964 | Pathologically | Population-based  cancer  registry | Healthy individuals  identified by  random digit dialing  and administration  lists matched by  age and sex | Population-based | PCR-RFLP  Masscode  system | 0.053 | NR |
| Liu et al. (2000)[^68^](#_ENREF_68) | Canada | CYP1A1  GSTM1  GSTT1 | Caucasian | NR | 149/103 | Pathologically | Nine district  inpatient and  outpatient  clinics | Healthy individuals  from spouse,  unrelated family  members, or no  family member and  by random digit  dialing | Hospital-based | PCR | NR | Drinking,  smoking, and  ethnicity |
| Barber et al.  (1999)[^69^](#_ENREF_69) | UK | TNFa TNFb | NR | NR | 64/101 | Pathologically (51)  Radiologically | University  hospital | Healthy blood donors | Population-based | PCR | 0.55 | NR |
| Bartsch et al.  (1998)[^70^](#_ENREF_70) | Germany | NAT1 NAT2  GSTM1 | Caucasian | NR | 81/78 | Pathologically | Three centers in  USA and five  in Europe | Healthy blood donors  and cancer free  patients | Hospital-based | PCR | 0.33 | NR |
| Lee et al.  (1997)[^71^](#_ENREF_71) | Korea | CYP1A1 | Asian | NR | 45/53 | Clinically  Radiologically  Pathologically | NR | Healthy individuals | Hospital-based | PCR | 0.44 | NR |

Abbreviations: ABO, chromosomes 9q34; ADH1B, alcohol dehydrogenase-1B; ALDH2, aldehyde dehydrogenase 2; APE1, apurinic/apyrimidinic endonuclease; ATM, ataxia-telangiectasia mutated; CAPN10, calpain-10; CLPTM1L-TERT, cleft lip and palate transmembrane 1-like-telomerase reverse transcriptase; COX-2, cyclooxygenase-2; CTLA-4, Cytotoxic T-lymphocyte antigen-4; CTNND, catenin delta 1; CYP1A1, cytochrome P-450 1A1; CYP2A13, cytochrome P4502A13; DDB2, DNA damage binding; ERCC1, excision repair cross complementation 1; CFTR, cystic fibrosis transmembrane conductance regulator; GSTM1, glutathione S-transferase mu 1; GSTP1, Glutathione S-transferase P1; GSTT1, Glutathione S-transferase theta 1; HIF-1, hypoxia-inducible factor-1; HOTAIR, HOX transcript antisense RNA; HOTTIP, HOXA transcript at the distal tip; HWE, Hardy–Weinberg equilibrium; IGF, insulin-like growth factors; IL-1b, Interleukin-1b; LIG3, Ligase 3; MDM2, mouse double minute 2 homologue; MTHFR, Methylenetetrahydrofolate reductase; MTR, methionine synthase; MTRR, methionine synthase reductase; NAT, N-acetyltransferase; NR, not reported; NR5A2, nuclear receptor subfamily 5, group A, member 2; OGG1, 8-oxoguanine DNA glycosylase; PARP1, poly(ADP-ribose) polymerase 1; PCR, polymerase chain reaction; PCR-RFLP, PCR-restriction fragment length polymorphisms; POLB, Port of Long Beach; SHH, Sonic Hedgehog; SPINK1, Kazal type 1 serine protease inhibitor; SSTR5, somatostatin receptor 5; STAT3, signal transducer and activator of transcription 3; TNFa, tumour necrosis factor alpha; TP53, Tumor protein p53; TRIB1, tribbles homolog 1 (Drosophila) gene; TS, thymidylate synthase; U2AF65, U2 small nuclear ribonucleoprotein auxiliary factor 65; UGT1A7, UDP glucuronosyltransferase 7; USA, United States of America; VDR, vitamin D receptor; XPA, xeroderma pigmentosum group A; XPC, xeroderma pigmentosum group C; XPD, xeroderma pigmentosum group D; XPF, xeroderma pigmentosum group F; XRCC1, X-ray repair cross-complementing group 1.

**Supplementary figures**


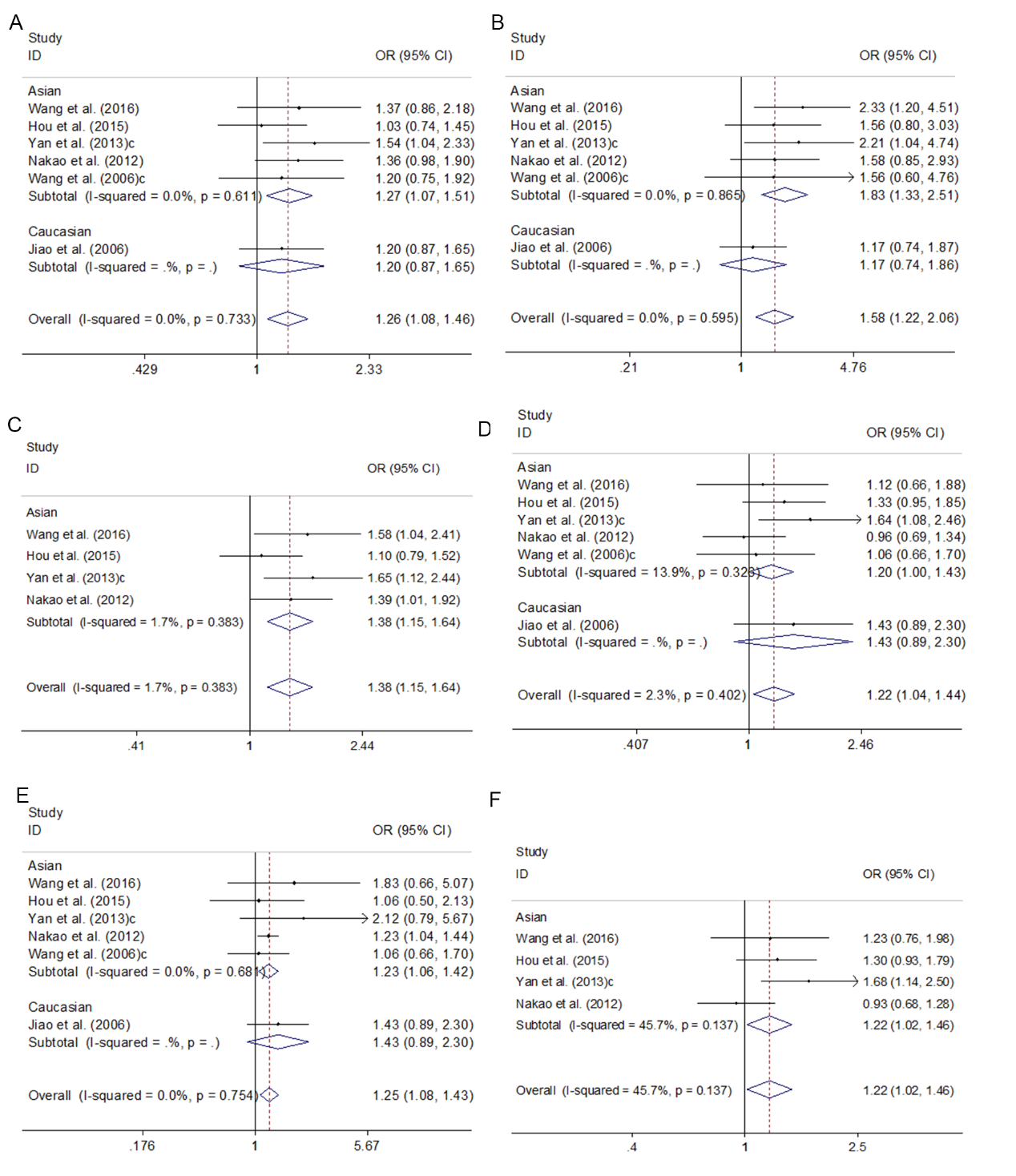


Supplmentary figure 1. Subgroup study of the association between XRCC1 Arg399GIn and XRCC1 Arg194Trp genetic polymorphisms and PC risk. A. XRCC1 Arg399GIn polymorphism (GA vs. GG); B. XRCC1 Arg399GIn polymorphism (AA vs. GG); C. XRCC1 Arg399GIn polymorphism (GA+AA vs. GG); D. XRCC1 Arg194Trp polymorphism (CT vs. CC); E. XRCC1 Arg194Trp polymorphism (TT vs. CC); F. XRCC1 Arg194Trp polymorphism (CT+TT vs. CC). Abbreviations: CI, confidence Interval; OR, odds ratio; PC, Pancreatic cancer; XRCC1, X-ray repair cross-complementing group 1 gene.


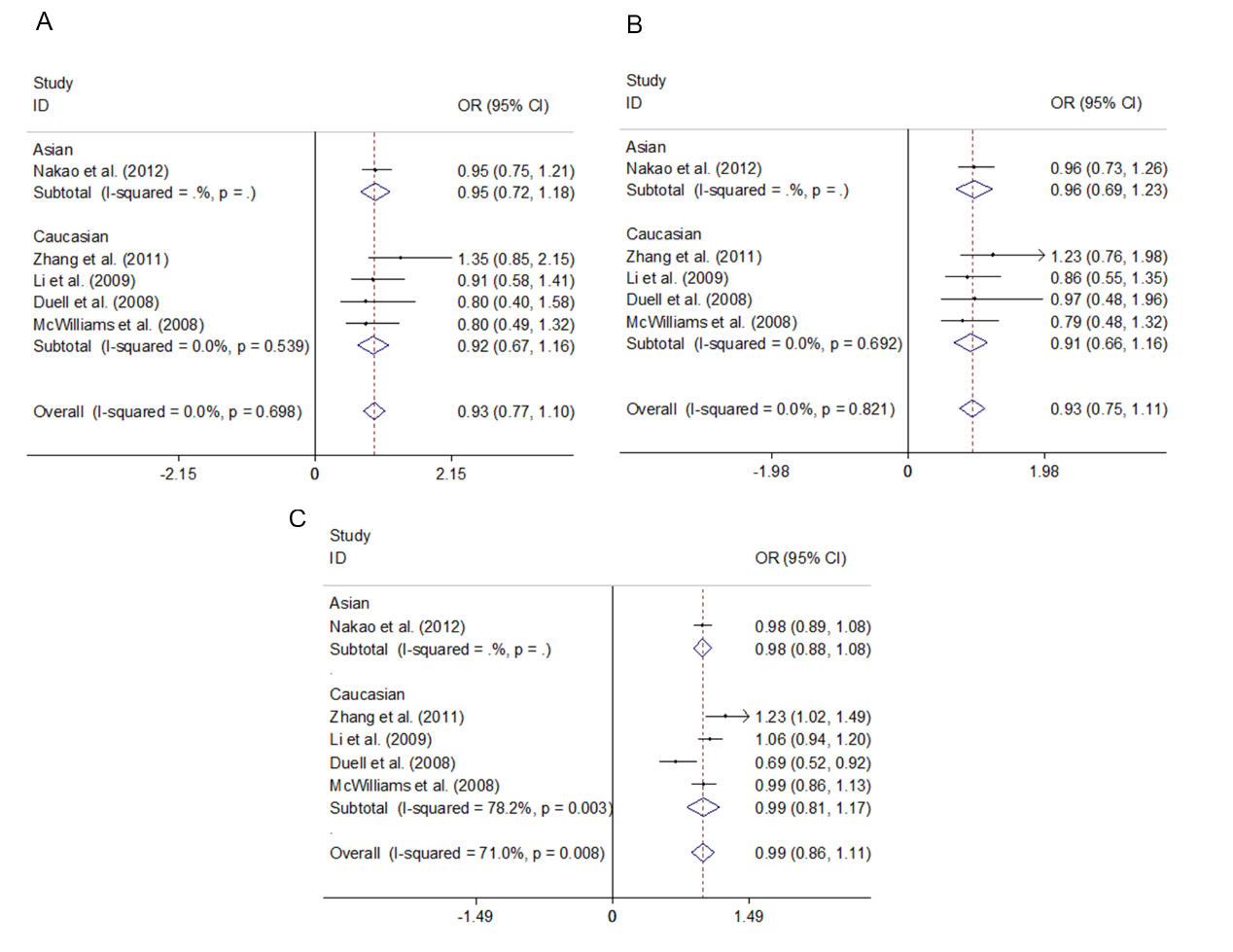


Supplmentary figure 2. Subgroup study of the association between OGG1 Ser326Cys polymorphism and PC risk. A. CC vs. GG; B. CC vs. GG+GC; C. CC+GC vs. GG. Abbreviations: CI, confidence Interval; OR, odds ratio; PC, Pancreatic cancer; OGG1, 8-oxoguanineDNA glycosylase 1.


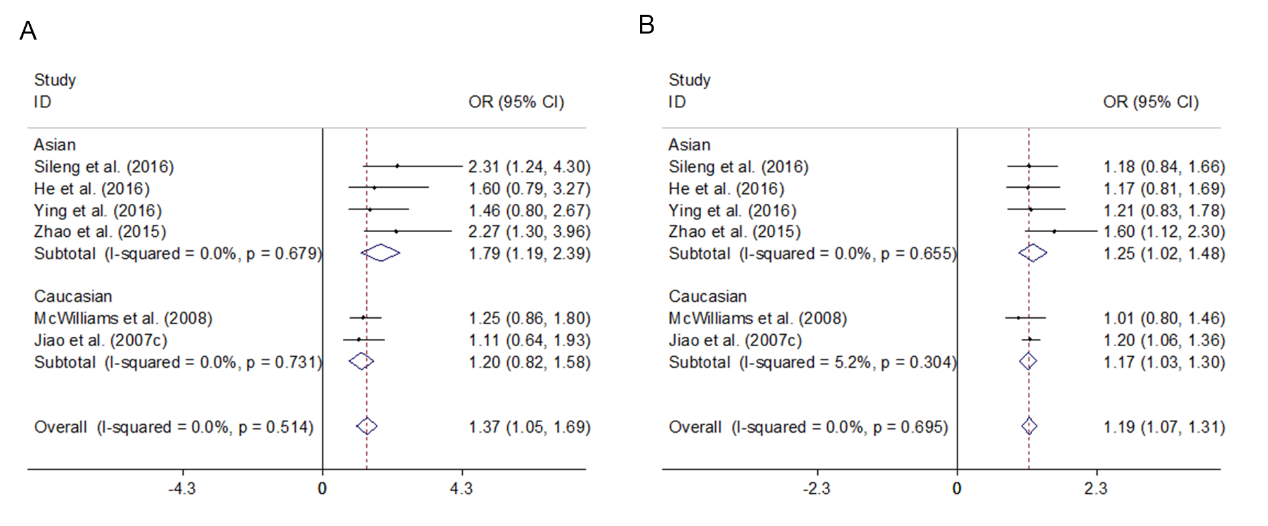


Supplmentary figure 3. Subgroup study of the association between ERCC2 rs13181 polymorphism and PC risk. A. CC vs. AA; B. AC/CC vs. AA. Abbreviations: CI, confidence Interval; OR, odds ratio; PC, Pancreatic cancer; ERCC2, excision repair cross complementation 2.

**Supplementary references**

1. Wu Y, Lu ZP, Zhang JJ, et al. Association between ERCC2 Lys751Gln polymorphism and the risk of pancreatic cancer, especially among Asians: evidence from a meta-analysis. Oncotarget 2017; **8**(30): 50124-32.

2. Wang W, Fei Y, Liu SL. CTNND1 755 T>G Promoter Polymorphism and Risk of Pancreatic Carcinoma in Chinese. Journal of clinical laboratory analysis 2017; **31**(3).

3. Hu P, Qiao O, Wang J, et al. rs1859168 A > C polymorphism regulates HOTTIP expression and reduces risk of pancreatic cancer in a Chinese population. World journal of surgical oncology 2017; **15**(1): 155.

4. Wang LJ, Wang HT, Wang XX. Association of XRCC1 gene polymorphisms and pancreatic cancer risk in a Chinese population. Genetics and molecular research : GMR 2016; **15**(2).

5. Hussain S, Bano R, Tahir Khan M, Haroon Khan M. Association of the CYP17-34T/C Polymorphism with Pancreatic Cancer Risk. Asian Pacific journal of cancer prevention : APJCP 2016; **17**(S3): 71-5.

6. Sileng A, Pan RH, Li GH, et al. ERCC1 rs3212986 and ERCC2 rs13181 gene polymorphisms contributes to the susceptibility to pancreatic cancer in a Chinese population. International journal of clinical and experimental pathology 2016; **9**(5): 5687-93.

7. He MG, Zheng K, Tan D, Wang ZX. Association between ERCC1 and ERCC2 gene polymorphisms and susceptibility to pancreatic cancer. Genetics and molecular research : GMR 2016; **15**(1).

8. Ying MF, Zhao R. Role of single nucleotide polymorphisms of DNA repair genes in susceptibility to pancreatic cancer in Chinese population. Genetics and molecular research : GMR 2016; **15**(1).

9. Zhao F, Shang Y, Zeng C, Gao D, Li K. Association of single nucleotide polymorphisms of DNA repair genes in NER pathway and susceptibility to pancreatic cancer. International journal of clinical and experimental pathology 2015; **8**(9): 11579-86.

10. Ueno M, Ohkawa S, Morimoto M, et al. Genome-wide association study-identified SNPs (rs3790844, rs3790843) in the NR5A2 gene and risk of pancreatic cancer in Japanese. Scientific reports 2015; **5**: 17018.

11. Hou BH, Jian ZX, Cui P, Li SJ, Tian RQ, Ou JR. Association and Intragenic Single-Nucleotide Polymorphism Interactions of the XRCC1 Polymorphisms for Pancreatic Cancer Susceptibility. Pancreas 2016; **45**(4): 546-51.

12. Qin L, Yu T. Association between rs9904341 G<C gene polymorphism and susceptibility to pancreatic cancer in a Chinese population. Genetics and molecular research : GMR 2015; **14**(2): 5197-202.

13. Li L, Shang F, Zhang W, et al. Role of vitamin D receptor gene polymorphisms in pancreatic cancer: a case-control study in China. Tumour biology : the journal of the International Society for Oncodevelopmental Biology and Medicine 2015; **36**(6): 4707-14.

14. Zhao ZM, Li CG, Hu MG, Zhao GD, Liu R. Association of c.461G>A genetic variant of OGG1 gene with pancreatic cancer susceptibility in Chinese. Genetics and molecular research : GMR 2014; **13**(3): 7256-61.

15. Tian J, Zhu B, Tian Y, Zhong R, Miao X, Wang L. [Association between pancreatic cancer risk and the interaction of U2AF65 gene polymorphisms and smoking]. Zhonghua liu xing bing xue za zhi = Zhonghua liuxingbingxue zazhi 2014; **35**(6): 710-3.

16. Lu XX, Hu JJ, Fang Y, et al. A case-control study indicates that the TRIB1 gene is associated with pancreatic cancer. Genetics and molecular research : GMR 2014; **13**(3): 6142-7.

17. Liu C, Wang Y, Huang H, et al. Association between CLPTM1L-TERT rs401681 polymorphism and pancreatic cancer risk among Chinese Han population. Tumour biology : the journal of the International Society for Oncodevelopmental Biology and Medicine 2014; **35**(6): 5453-7.

18. Chen H, Zhou B, Lan X, Wei D, Yuan T, Chen P. Association between single-nucleotide polymorphisms of OGG1 gene and pancreatic cancer risk in Chinese Han population. Tumour biology : the journal of the International Society for Oncodevelopmental Biology and Medicine 2014; **35**(1): 809-13.

19. Chen H, Tang C, Liu M, et al. Association of XRCC1 gene single nucleotide polymorphisms and susceptibility to pancreatic cancer in Chinese. Tumour biology : the journal of the International Society for Oncodevelopmental Biology and Medicine 2014; **35**(1): 27-32.

20. Yan D, Wang XY, Li HJ, Xu XJ, Zhu GB, He TY. [Relationship between single nucleotide polymorphisms and its haplotype of X-ray repair cross complementing group 1 and susceptibility of pancreatic carcinoma]. Zhonghua zhong liu za zhi [Chinese journal of oncology] 2013; **35**(6): 472-7.

21. Xiang H, Zhao W, Sun Y, et al. CD86 gene variants and susceptibility to pancreatic cancer. Journal of cancer research and clinical oncology 2012; **138**(12): 2061-7.

22. Lang C, Chen L, Li S. Cytotoxic T-lymphocyte antigen-4 +49G/A polymorphism and susceptibility to pancreatic cancer. DNA and cell biology 2012; **31**(5): 683-7.

23. Nakao M, Hosono S, Ito H, et al. Selected polymorphisms of base excision repair genes and pancreatic cancer risk in Japanese. Journal of epidemiology 2012; **22**(6): 477-83.

24. Willis JA, Olson SH, Orlow I, et al. A replication study and genome-wide scan of single-nucleotide polymorphisms associated with pancreatic cancer risk and overall survival. Clinical cancer research : an official journal of the American Association for Cancer Research 2012; **18**(14): 3942-51.

25. Dong X, Li Y, Tang H, et al. Insulin-like growth factor axis gene polymorphisms modify risk of pancreatic cancer. Cancer epidemiology 2012; **36**(2): 206-11.

26. Wang X, Liu Y, Ren H, et al. Polymorphisms in the hypoxia-inducible factor-1alpha gene confer susceptibility to pancreatic cancer. Cancer biology & therapy 2011; **12**(5): 383-7.

27. Rizzato C, Campa D, Giese N, et al. Pancreatic cancer susceptibility loci and their role in survival. PloS one 2011; **6**(11): e27921.

28. Li D, Tanaka M, Brunicardi FC, Fisher WE, Gibbs RA, Gingras MC. Association between somatostatin receptor 5 gene polymorphisms and pancreatic cancer risk and survival. Cancer 2011; **117**(13): 2863-72.

29. Sonoyama T, Sakai A, Mita Y, et al. TP53 codon 72 polymorphism is associated with pancreatic cancer risk in males, smokers and drinkers. Molecular medicine reports 2011; **4**(3): 489-95.

30. Zhang J, Zhang X, Dhakal IB, Gross MD, Kadlubar FF, Anderson KE. Sequence variants in antioxidant defense and DNA repair genes, dietary antioxidants, and pancreatic cancer risk. International journal of molecular epidemiology and genetics 2011; **2**(3): 236-44.

31. Fong PY, Fesinmeyer MD, White E, et al. Association of diabetes susceptibility gene calpain-10 with pancreatic cancer among smokers. Journal of gastrointestinal cancer 2010; **41**(3): 203-8.

32. Fei Y, Hu J, Liu S, Liu X, Wang F, Gong J. E-cadherin-160 C/A promoter polymorphism and risk of pancreatic carcinoma in Chinese population. Cancer genetics and cytogenetics 2010; **197**(1): 25-31.

33. Naccarati A, Pardini B, Polakova V, et al. Genotype and haplotype analysis of TP53 gene and the risk of pancreatic cancer: an association study in the Czech Republic. Carcinogenesis 2010; **31**(4): 666-70.

34. Petersen GM, Amundadottir L, Fuchs CS, et al. A genome-wide association study identifies pancreatic cancer susceptibility loci on chromosomes 13q22.1, 1q32.1 and 5p15.33. Nature genetics 2010; **42**(3): 224-8.

35. Mohelnikova-Duchonova B, Vrana D, Holcatova I, Ryska M, Smerhovsky Z, Soucek P. CYP2A13, ADH1B, and ADH1C gene polymorphisms and pancreatic cancer risk. Pancreas 2010; **39**(2): 144-8.

36. Vrana D, Pikhart H, Mohelnikova-Duchonova B, et al. The association between glutathione S-transferase gene polymorphisms and pancreatic cancer in a central European Slavonic population. Mutation research 2009; **680**(1-2): 78-81.

37. Hamacher R, Diersch S, Scheibel M, et al. Interleukin 1 beta gene promoter SNPs are associated with risk of pancreatic cancer. Cytokine 2009; **46**(2): 182-6.

38. Rafnar T, Sulem P, Stacey SN, et al. CLPTM1L-TERT. Nature genetics 2009; **41**(2): 221-7.

39. Li D, Suzuki H, Liu B, et al. DNA repair gene polymorphisms and risk of pancreatic cancer. Clinical cancer research : an official journal of the American Association for Cancer Research 2009; **15**(2): 740-6.

40. Suzuki H, Li Y, Dong X, Hassan MM, Abbruzzese JL, Li D. Effect of insulin-like growth factor gene polymorphisms alone or in interaction with diabetes on the risk of pancreatic cancer. Cancer epidemiology, biomarkers & prevention : a publication of the American Association for Cancer Research, cosponsored by the American Society of Preventive Oncology 2008; **17**(12): 3467-73.

41. Xu DK, Zhang XM, Zhao P, et al. [Association between single nucleotide polymorphisms in the promoter of cyclooxygenase COX-2 gene and hereditary susceptibility to pancreatic cancer]. Zhonghua yi xue za zhi 2008; **88**(28): 1961-5.

42. Suzuki T, Matsuo K, Sawaki A, et al. Alcohol drinking and one-carbon metabolism-related gene polymorphisms on pancreatic cancer risk. Cancer epidemiology, biomarkers & prevention : a publication of the American Association for Cancer Research, cosponsored by the American Society of Preventive Oncology 2008; **17**(10): 2742-7.

43. Asomaning K, Reid AE, Zhou W, et al. MDM2 promoter polymorphism and pancreatic cancer risk and prognosis. Clinical cancer research : an official journal of the American Association for Cancer Research 2008; **14**(12): 4010-5.

44. Duell EJ, Bracci PM, Moore JH, Burk RD, Kelsey KT, Holly EA. Detecting pathway-based gene-gene and gene-environment interactions in pancreatic cancer. Cancer epidemiology, biomarkers & prevention : a publication of the American Association for Cancer Research, cosponsored by the American Society of Preventive Oncology 2008; **17**(6): 1470-9.

45. Ohnami S, Sato Y, Yoshimura K, et al. His595Tyr polymorphism in the methionine synthase reductase (MTRR) gene is associated with pancreatic cancer risk. Gastroenterology 2008; **135**(2): 477-88.

46. Zhang D, Zhou Y, Wu L, et al. Association of IL-6 gene polymorphisms with cachexia susceptibility and survival time of patients with pancreatic cancer. Annals of clinical and laboratory science 2008; **38**(2): 113-9.

47. Jiao L, Hassan MM, Bondy ML, et al. XRCC2 and XRCC3 gene polymorphism and risk of pancreatic cancer. The American journal of gastroenterology 2008; **103**(2): 360-7.

48. Kanda J, Matsuo K, Suzuki T, et al. Impact of alcohol consumption with polymorphisms in alcohol-metabolizing enzymes on pancreatic cancer risk in Japanese. Cancer science 2009; **100**(2): 296-302.

49. McWilliams RR, Bamlet WR, Cunningham JM, et al. Polymorphisms in DNA repair genes, smoking, and pancreatic adenocarcinoma risk. Cancer research 2008; **68**(12): 4928-35.

50. Jiao L, Doll MA, Hein DW, et al. Haplotype of N-acetyltransferase 1 and 2 and risk of pancreatic cancer. Cancer epidemiology, biomarkers & prevention : a publication of the American Association for Cancer Research, cosponsored by the American Society of Preventive Oncology 2007; **16**(11): 2379-86.

51. Jiao L, Bondy ML, Hassan MM, et al. Glutathione S-transferase gene polymorphisms and risk and survival of pancreatic cancer. Cancer 2007; **109**(5): 840-8.

52. Jiao L, Hassan MM, Bondy ML, Abbruzzese JL, Evans DB, Li D. The XPD Asp312Asn and Lys751Gln polymorphisms, corresponding haplotype, and pancreatic cancer risk. Cancer letters 2007; **245**(1-2): 61-8.

53. Duell EJ, Casella DP, Burk RD, Kelsey KT, Holly EA. Inflammation, genetic polymorphisms in proinflammatory genes TNF-A, RANTES, and CCR5, and risk of pancreatic adenocarcinoma. Cancer epidemiology, biomarkers & prevention : a publication of the American Association for Cancer Research, cosponsored by the American Society of Preventive Oncology 2006; **15**(4): 726-31.

54. Jiao L, Bondy ML, Hassan MM, et al. Selected polymorphisms of DNA repair genes and risk of pancreatic cancer. Cancer detection and prevention 2006; **30**(3): 284-91.

55. Piepoli A, Gentile A, Valvano MR, et al. Lack of association between UGT1A7, UGT1A9, ARP, SPINK1 and CFTR gene polymorphisms and pancreatic cancer in Italian patients. World journal of gastroenterology 2006; **12**(39): 6343-8.

56. Wang L, Lin DX, Lu XH, Miao XP, Li H. [Polymorphisms of the DNA repair genes XRCC1 and XPC: relationship to pancreatic cancer risk]. Wei sheng yan jiu = Journal of hygiene research 2006; **35**(5): 534-6.

57. Lempinen M, Paju A, Kemppainen E, et al. Mutations N34S and P55S of the SPINK1 gene in patients with chronic pancreatitis or pancreatic cancer and in healthy subjects: a report from Finland. Scandinavian journal of gastroenterology 2005; **40**(2): 225-30.

58. Li D, Ahmed M, Li Y, et al. 5,10-Methylenetetrahydrofolate reductase polymorphisms and the risk of pancreatic cancer. Cancer epidemiology, biomarkers & prevention : a publication of the American Association for Cancer Research, cosponsored by the American Society of Preventive Oncology 2005; **14**(6): 1470-6.

59. Matsubayashi H, Skinner HG, Iacobuzio-Donahue C, et al. Pancreaticobiliary cancers with deficient methylenetetrahydrofolate reductase genotypes. Clinical gastroenterology and hepatology : the official clinical practice journal of the American Gastroenterological Association 2005; **3**(8): 752-60.

60. Miyasaka K, Kawanami T, Shimokata H, Ohta S, Funakoshi A. Inactive aldehyde dehydrogenase-2 increased the risk of pancreatic cancer among smokers in a Japanese male population. Pancreas 2005; **30**(2): 95-8.

61. Verlaan M, Drenth JP, Truninger K, et al. Polymorphisms of UDP-glucuronosyltransferase 1A7 are not involved in pancreatic diseases. Journal of medical genetics 2005; **42**(10): e62.

62. Wang L, Miao X, Tan W, et al. Genetic polymorphisms in methylenetetrahydrofolate reductase and thymidylate synthase and risk of pancreatic cancer. Clinical gastroenterology and hepatology : the official clinical practice journal of the American Gastroenterological Association 2005; **3**(8): 743-51.

63. Beranek H, Teich N, Witt H, Schulz HU, Mossner J, Keim V. Analysis of tumour necrosis factor alpha and interleukin 10 promotor variants in patients with chronic pancreatitis. European journal of gastroenterology & hepatology 2003; **15**(11): 1223-7.

64. Matsubayashi H, Fukushima N, Sato N, et al. Polymorphisms of SPINK1 N34S and CFTR in patients with sporadic and familial pancreatic cancer. Cancer biology & therapy 2003; **2**(6): 652-5.

65. Teich N, Schulz HU, Witt H, Bohmig M, Keim V. N34S, a pancreatitis associated SPINK1 mutation, is not associated with sporadic pancreatic cancer. Pancreatology : official journal of the International Association of Pancreatology (IAP) [et al] 2003; **3**(1): 67-8.

66. Duell EJ, Holly EA, Bracci PM, Liu M, Wiencke JK, Kelsey KT. A population-based, case-control study of polymorphisms in carcinogen-metabolizing genes, smoking, and pancreatic adenocarcinoma risk. Journal of the National Cancer Institute 2002; **94**(4): 297-306.

67. Duell EJ, Holly EA, Bracci PM, Wiencke JK, Kelsey KT. A population-based study of the Arg399Gln polymorphism in X-ray repair cross- complementing group 1 (XRCC1) and risk of pancreatic adenocarcinoma. Cancer research 2002; **62**(16): 4630-6.

68. Liu G, Ghadirian P, Vesprini D, et al. Polymorphisms in GSTM1, GSTT1 and CYP1A1 and risk of pancreatic adenocarcinoma. British journal of cancer 2000; **82**(10): 1646-9.

69. Barber MD, Powell JJ, Lynch SF, Fearon KC, Ross JA. A polymorphism of the interleukin-1 beta gene influences survival in pancreatic cancer. British journal of cancer 2000; **83**(11): 1443-7.

70. Bartsch H, Malaveille C, Lowenfels AB, Maisonneuve P, Hautefeuille A, Boyle P. Genetic polymorphism of N-acetyltransferases, glutathione S-transferase M1 and NAD(P)H:quinone oxidoreductase in relation to malignant and benign pancreatic disease risk. The International Pancreatic Disease Study Group. European journal of cancer prevention : the official journal of the European Cancer Prevention Organisation (ECP) 1998; **7**(3): 215-23.

71. Lee HC, Yoon YB, Kim CY. Association between genetic polymorphisms of the cytochromes P-450 (1A1, 2D6, and 2E1) and the susceptibility to pancreatic cancer. The Korean journal of internal medicine 1997; **12**(2): 128-36.
